# Immunoglobulin E-Mediated Allergy Plays a Role in Atopic Eczema as Shown in the Atopy Patch Test

**DOI:** 10.1097/WOX.0b013e3181661472

**Published:** 2008-03-15

**Authors:** Ulf Darsow, Johannes Ring

**Affiliations:** 1 Department of Dermatology and Allergy Biederstein, Division of Environmental Dermatology and Allergy GSF/TUM, Technical University Munich, Biedersteiner Straße 29, D-80802 München, Germany

**Keywords:** IgE, diagnostic techniques, atopy patch test, atopic eczema

## Abstract

Although the pathophysiology of immunoglobulin E (IgE)-mediated allergic rhinoconjunctivitis and bronchial asthma is rather well established, the role of allergy in atopic eczema (AE) is still controversial. By a technique called atopy patch test, aeroallergens like house dust mite, animal dander, or pollen were proven as relevant trigger factors in a subgroup of patients with AE. The atopy patch test is an epicutaneous patch test with such allergens known to elicit IgE-mediated reactions, and used for the evaluation of eczematous skin reactions. In a series of single-center and multicenter studies, a method was developed, standardized, and compared with other diagnostic techniques (radioallergosorbent test, skin prick test) in AE patients. With regard to clinical history, the most specific results were obtained with the atopy patch test (allergen-dependent, 69%-92%), whereas sensitivity was higher for skin prick test (range, 69%-82%) and specific IgE (range, 65%-94%). The characterization of a patient subgroup with relevant IgE-mediated allergy may lead to more efficient avoidance and eventually even specific immunotherapy strategies in the management of AE.

## 

Atopic eczema (AE, atopic dermatitis, AE/dermatitis syndrome) is a clinically well-defined inflammatory, chronically relapsing, highly pruritic skin disease with a typically age-related distribution and morphology[1-3] and a prevalence of 2% to 10% in the population [1,4,5]. Elevated immunoglobulin E (IgE) production, especially against aeroallergens and food allergens, and/or altered unspecific reactivity are frequent findings in patients with AE and concomitant respiratory atopic diseases [6,7]. As a multifactorial disease with a genetic background, AE has a large number of individually different trigger factors [8-11].

The deterioration of AE skin lesions in some patients after contact with certain IgE-inducing allergens like house dust mite, pollen, or animal dander is an old clinical observation. Consequently, allergen avoidance strategies have been used to improve the course of AE in some studies [12-17].

The inflammatory infiltrate of AE lesions consists to a large proportion of CD4^+ ^T helper (T_H_) cells. High IgE production in patients with AE is explained by an impaired balance of the T-cell populations T_H_1 and T_H_2, with a predominance of interleukin-4- and interleukin-13-producing T_H_2 cells [18-23]. Aeroallergens are able to penetrate the disturbed skin barrier[24] in patients with AE and were found in direct contact with antigen-presenting Langerhans cells [25]. The discovery of IgE and IgE-binding structures on the surface of epidermal Langerhans cells[26-29] resulted in a new concept that allergy contributes to the pathophysiology of AE because all of the major components of an IgE-mediated reaction are present in the epidermis. Subsequently, the function of IgE in antigen presentation was shown by Maurer et al.[30] However, the question whether allergy plays a role in practice still remained: measurement of specific serum IgE and skin prick tests or intracutaneous injections of allergen solutions are clinical routine to diagnose IgE-mediated sensitizations,[6,31] but in AE, they reveal often multiple sensitizations without clinical relevance. Furthermore, the morphology of skin test reactions (wheal and flare) does not resemble the clinical manifestation of AE, nor do they represent the appropriate dimensions of the skin immune system. An additional diagnostic tool for aeroallergen-triggered AE was needed, and the proof of concept study was done with a procedure our group called atopy patch test (APT) [32].

Rostenberg and Sulzberger[33] described in 1937 a series of 12,000 patch tests with a wide variety of allergens, including aeroallergens in different patient groups. In 1982, Mitchell et al[34] published the first experimental patch test with aeroallergens for patients with AE. By others, eczematous reactions could be elicited with different methods, but the methodology and definition of positive reactions in these trials were not comparable [35-45]. No clear correlation with history was obtained in larger groups of patients. Potentially irritating procedures like stratum corneum abrasion,[34,46,47] tape stripping,[48-50] or addition of sodium laurylsulfate[51] were necessary to enhance allergen penetration. In 1989, the term atopy patch test was proposed with the following definition: an epicutaneous patch test with allergens known to elicit IgE-mediated reactions, and the evaluation of eczematous skin reactions after 48 and 72 hours [32,52]. The first effort was to standardize APT and possibly develop a method for clinical routine giving positive results only in patients with AE and showing significant concordance to clinical relevance parameters of aeroallergen allergy.

## Methodological studies

**Figure 1:**
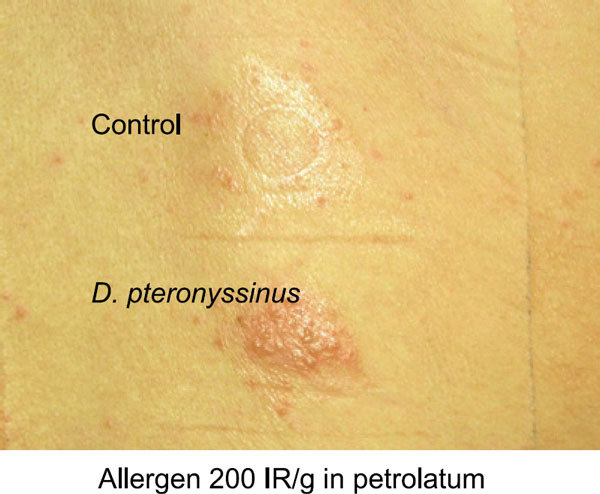
**The APT with house dust mite *D. pteronyssinus *in a patient with AE**. Eczematous reaction after 48 hours.

Allergen lyophilisates of house dust mite *Dermatophagoides pteronyssinus*, cat dander, grass pollen, and in later studies, of birch and mugwort pollen were used. The test preparations were developed on a noncommercial basis in cooperation with several industrial allergen suppliers to maintain a high standard of batch stability and reproducibility (Hermal and Allergopharma, Reinbek, Germany; Stallergénes, Antony, France). Application in large aluminum Finn chambers (12-mm diameter) on clinically uninvolved, nonabraded, and untreated back skin was superior to the use of small Finn chambers. Reproducibility of elicited APT reactions within a mean of 16 months was 94%. Vehicles were tested in control areas in all patients and remained in general negative. At the beginning, grading of positive APT reactions was done after 48 and 72 hours according to the International Contact Dermatitis Reseach Group rules [53,54]. Only reactions with infiltration were regarded as clear-cut positive (example in Figure 1). All APT studies were performed after discontinuance of systemic antihistamines (the effect of antihistamines on APT is not known to date) and systemic and topical (test area) steroids for at least 7 days.

### Role of the vehicle

In a pilot study[55] involving 36 patients with AE, the reactions of 17 patients (47%) were graded as clear-cut positive. Control sites (petrolatum, hydrogel) remained negative, non-atopic volunteers and patients with respiratory atopy (allergic rhinoconjunctivitis) only were also negative in APT. Allergens in petrolatum vehicle elicited twice as many positive APT reactions as the same dose in a hydrogel. Thirty-six percent of patients reacted to house dust mite *D. pteronyssinus*, 22% to cat dander, and 16% to grass pollen. A D. *pteronyssinus*-positive APT was accompanied in 77% by a corresponding elevated specific IgE (skin prick test, 62%).

### Dose-response effects and role of localization of eczema

Allergen concentrations of 500, 3000, 5000, and 10,000 protein nitrogen units (PNU)/g in petrolatum were compared in another study[56] in 57 patients. The frequency of clear-cut positive APT reactions was significantly higher in patients with eczematous skin lesions in air-exposed areas (69%) as compared with patients without this predictive pattern (39%; *P *= 0.02). In the first group, the maximum APT reactivity was reached at a lower allergen dose of around 5000 PNU/g.

Two hundred fifty-three adult patients with AE (Table 1) participated in a randomized, double-blind, multicenter study on dose-response, safety, and clinical covariates of the APT [57]. The allergen dose with the most clear-cut results (positive or negative) in adults was found for *D. pteronyssinus*, cat dander, and grass pollen between 5000 and 7000 PNU/g. Most patients reacted only to 1 allergen, rarely to 2 or 3.

**Table 1:** Clinical Covariates of the APT in 2 Multicenter Studies With Different Allergen Standardization

	Skin Prick		sIgE		APT		History	
				
	A	B	A	B	A	B	A	B
*D. pteronyssinus *	59	56	56	56	34	39	52	34
Cat dander	54	44	49	46	12	10	23	30
Grass pollen	65	57	75	59	18	15	33	31
Birch pollen	65	49	65	53	11	17	13	20

A indicates German multicenter[57] (7 centers, N = 253 adults, 3000-10,000 PNU/g); B, European multicenter[59] (6 countries, 12 centers, N = 314, 200 IR/g). Values are expressed as percentages.

### Age

In 30 children and adolescents 14 years old or younger with AE enrolled in a double-blind dose-response multicenter study,[58] a lower frequency of positive APT reactions compared with adults was seen for *D. pteronyssinus *(34% vs 41% in adults) and cat dander (12% vs 17%). For *D. pteronyssinus *and grass pollen, lower allergen doses for APT seem possible in children because maximal response rates were obtained for these allergens with 3000 PNU/g, half of the adult allergen concentration.

### Allergen standardization

Comparing different allergen standardization systems in 50 patients with parallel testing, the allergen doses of 7000 PNU/g and 200 IR/g (biological unit) were found to have similar concordance with the patients' clinical history: 71% to 73% of APTwere corroborated by a corresponding positive or negative history of AE flares after contact with the specific allergen. Expressed as major allergen content, 200 IR/g correspond to 59 μg/mL *Der p1*, 9 μg/mL *Fel d1*, or 2 μg/mL *Phl p1*.

In summary, these studies showed that:

• a safe standardized APT method with positive reactions only in patients with AE was developed;

• allergen lyophilisate in petrolatum is the preferred galenic preparation;

• APT is possible on nonabraded skin without manipulation of the skin barrier function;

• allergen concentrations higher than in most prick test solutions are necessary for APT, but lower doses can be used in children;

• *D. pteronyssinus *is the most frequent allergen eliciting positive APT reactions, with reactions to pollen allergens also being very frequent; and

• high allergen-specific IgE in serum is not a prerequisite for a positive APT.

## APT and specific IgE

**Figure 2:**
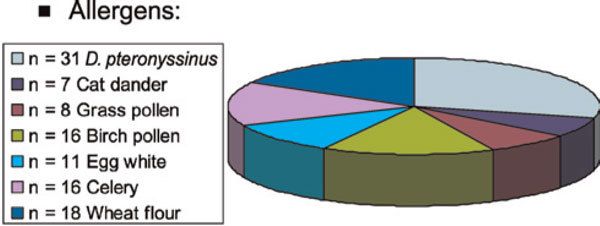
**In 53 (17%) of 314 patients of a European multicenter study**,[59]**positive APT reactions, but negative corresponding skin prick test/specific IgE results, were observed (1 allergen, n = 26; 2 allergens, n = 12)**. In 22 of these patients with a clear-cut positive APT result, no positive skin prick test or elevated specific serum IgE of the investigated allergen panel was seen (7% of total). The figure shows that all allergens contribute to these reactions.

The percentages of positive reactions in different test systems for IgE-mediated hypersensitivity obtained from our multicenter studies are given in Table 1. These and previous APT studies showed that positive APT occurred less frequently than positive skin prick tests or radioallergosorbent tests (RASTs) to the same allergen. Logistic regression analysis[57] revealed patient's history, skin prick test, and specific corresponding IgE for *D. pteronyssinus*, cat dander, and grass pollen as most important significant predictors of a positive APT (*P *< 0.001). However, the cross-tabulation also confirmed that high allergen-specific IgE in serum is not mandatory for a positive APT (example in Table 2), the same holds true for the correlation with skin prick tests. A European multicenter study[59] on standardized APT in 6 countries (n = 314) showed a subgroup of 7% APT-positive AE patients without any positive skin prick test or elevated specific IgE in the investigated allergen panel (Figure 2). Nevertheless, these reactions can be of clinical relevance and immunological specificity [60]. In conclusion,

• the APT may give further diagnostic information in addition to patient's history and classical tests of IgE-mediated hypersensitivity;

• the role for IgE in the reaction mechanism of APT is corroborated because in most APT-positive patients, elevated specific IgE was found compared with those with negative APT; and

• a cellular mechanism without direct involvement of IgE may be hypothesized to explain the clear-cut positive APT reactions in a subgroup of AE patients.

**Table 2:** Cross-Tabulation of APT and Specific IgE to House Dust Mite *D. pteronyssinus*, Results of a Multicenter Study[57]

APT 48-h Results in Adult Patients for House Dust Mite *D. pteronyssinus*	sIgE Negative	sIgE Positive	Total
APT negative	49	29	78
APT positive	13	60	73
Total	62	89	151

## IgE-mediated sensitization and APT: diagnostic precision

Unlike in food allergic patients, a "golden standard" of provocation of aeroallergen-induced AE is not established. The prospectively obtained history of allergen-induced exacerbations of AE, especially in a seasonal allergen, can be used to evaluate the clinical relevance of an APT result like in conventional patch testing. Rajka[3] reported on the phenomenon of "summer eruption," that is, eczema flares during spring and summer, the pollen seasons of birch and grass, in one third of patients with AE. According to our results, one third of patients with specific IgE to grass pollen can have a positive APT reaction to this allergen [57]. In a study on the influence of grass pollen on AE,[61] we tested 79 patients with an APT with 10,000 PNU/g grass pollen allergen mixture in petrolatum and simultaneously with 10 mg of dry unprocessed grass pollen of *Dactylis glomerata*. Significantly higher frequencies of positive APT occurred in patients with a history of exacerbation of AE in the summer months of the previous year or in direct contact with grass (n = 12, 75% had positive APT) compared with patients without this history (n = 67, 16% had positive APT; *P *< 0.001). Sensitivity and specificity of APT and classical tests for IgE-mediated sensitization are given in Table 3. The standardized APT also correlated with a predictive eczema pattern, skin prick test, and specific IgE to grass pollen (*P *< 0.01). Moreover, unprocessed grass pollen also elicited eczematous skin reactions on nonpretreated skin of patients with AE, significantly associated to history and a positive standardized APT with lyophilisate. Again, in healthy and rhinoconjunctivitis controls, no positive reactions were observed.

**Table 3:** Sensitivity and Specificity of Different Diagnostic Methods in 2 Studies With Patients With AE

Test	Sensitivity*	Specificity*
Single-center study, n = 79 (allergen, grass pollen)		
Skin prick	100	33
RAST	92	33
APT	75	84
APT multicenter study, n = 253 (3 allergens)		
Skin prick	69-82	44-53
RAST	65-94	42-64
APT	42-56	69-92

Better results are obtained with a seasonal allergen. Data from Ref. [61] and Ref. [57]

*Referring to predictive history of eczema exacerbations in pollen season or in direct contact with allergen, excluding questionable cases, depending on allergen.

Values are expressed as percentages.

In a larger patient group in the German multicenter study,[57] APT results of *D. pteronyssinus*, cat dander, and grass pollen were also statistically significantly associated with clinical history (*P *< 0.001, *χ*^2 ^and logistic regression; birch pollen, *P *= 0.1). Thus, sensitivity and specificity of different diagnostic tests could be compared. Allergen-dependent, the APT showed a higher specificity with regard to clinical relevance of an allergen than skin prick test and specific IgE, but also in most allergens, a lower sensitivity (Table 3). In a subgroup of these patients, specific activation and proliferation of T cells in peripheral blood was compared with the patient's APT result [62]. Positive APT reactions were significantly more frequent in patients with elevated CD54^+ ^or CD30^+ ^T cells after in vitro stimulation with the corresponding allergen. In addition, positive APT results were associated with an allergen-specific lymphocyte proliferation (*P *< 0.001). Positive APT reactions were not associated with disease severity in the SCORAD (scoring atopic dermatitis) system [62,63].

From APT biopsies, allergen-specific T cells have been cloned. In serial biopsies, T cells showed a characteristic T_H_2 secretion pattern (interleukin-4, -13) at 24 hours, whereas after 48 hours, a T_H_1 pattern (interferon-γ) like in chronic AE lesions was predominant [64-66]. Taken together, these findings:

• argue against the interpretation of APT results as irritative or nonspecific;

• suggest that pollen are involved in AE flares in some patients previously diagnosed as having UV-triggered eczema;

• demonstrate the clinical relevance of positive APT reactions and the different compartments of allergic inflammation that can be investigated with skin prick test, specific serum IgE determination, and APT;

• show that allergen-specific T cells and IgE play a role in the pathophysiology of APT reactions; and

• sustain the concept that AE is not only a disease of dry skin or barrier dysfunction, but also an allergic disease.

## The future of the APT

**Figure 3:**
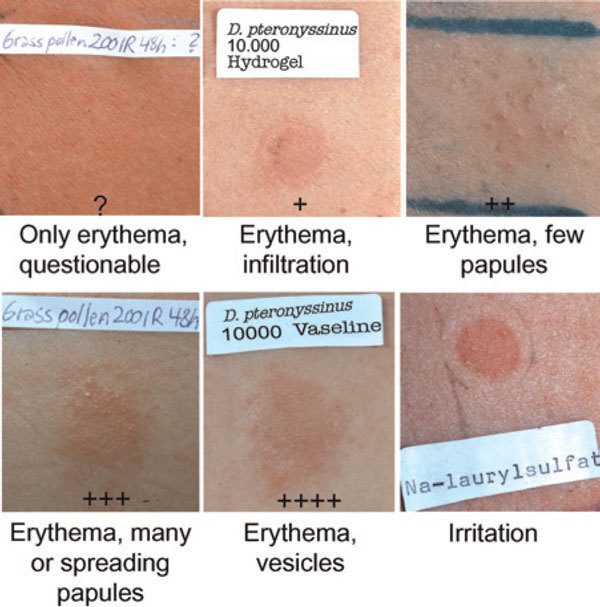
**The APT reaction grading key (2003 European Task Force on Atopic Dermatitis consensus)**.

Appropriate allergen-specific avoidance strategies[13,14,16,67,68] are recommended in patients showing positive APT reactions. The identified subgroup of patients may profit extraordinarily from allergen avoidance, but controlled studies using specific provocation and elimination procedures in patients with positive and negative APT results are still necessary. Standardization of major allergen content and achieving an increase in the sensitivity of APT are important goals of ongoing trials. The combination of APT and specific IgE results may lead to higher diagnostic precision, but the problem of discordant tests has to be solved. Meetings of most European groups performing APT for clinical use in 1997, 1998, and 2003 resulted in a consensus APT reading key of the European Task Force on Atopic Dermatitis (Figure 3) [60,69]. The European multicenter trial also started the investigation of food allergen patch testing with lyophilisates in petrolatum. The development of a standardized APT for food allergy is a future plan, as well as a study on the mechanisms of APT reactions involving both patients with intrinsic and extrinsic AE and investigating the role of the Fc*ε *receptor I and allergen-specific T-cell populations. Furthermore, the APT will be used in trials on topical and systemic therapy for AE and is evaluated as itch model [70,71]. Other aeroallergens of regional significance are to be standardized. A test for the clinical relevance of an aeroallergen sensitization that can be applied in the allergist's practice may evolve soon. The APT may also be valuable in selecting patients for specific immunotherapy. To solve the remaining tasks in due time and to overcome the methodological inconsistencies of different groups, the organization of international multicenter studies is necessary. A recent European Academy of Allergology and Clinical Immunology/Global Allergy and Asthma European Network position paper focused on clinical results and unresolved problems of APT [72].

## Concluding remarks

The described APT methodology was evaluated in several hundreds of patients with AE. In a large subgroup of them, IgE-dependent allergic reactions that are elicited by the transdermal route play a pathophysiological role. For patients with aeroallergen-triggered disease, the APT may provide an important diagnostic tool, a provocation test of the skin in analogy to the specific provocation methods in respiratory atopy. As in respiratory atopy, the results of our studies sustain B. Wüthrich's concept of extrinsic/allergic versus intrinsic/idiopathic AE [73].

Positive APT results were obtained in some AE patients with negative skin prick tests and RAST, but predictive history. According to the previously mentioned concept, these cases may also be classified as "extrinsic," and with the back-ground of the recently proposed novel nomenclature for allergy,[74] we suggested to diagnose these cases as "non-IgE-associated AE (dermatitis syndrome)."[60]

## References

[B1] Hanifin JM Rajka G Diagnostic features of atopic dermatitis Acta Derm Venereol 1980 1 146 148

[B2] Jones HE Inouye JC McGerity JL Lewis CW Atopic disease and serum immunoglobulin-E Br J Dermatol 1975 1 17 25 1156542 10.1111/j.1365-2133.1975.tb03028.x

[B3] Rajka G Essential Aspects of Atopic Dermatitis 1989 Berlin: Springer

[B4] Hanifin JM Clinical and basic aspects of atopic dermatitis Semin Dermatol 1983 1 5

[B5] Leung DYM Rhodes AR Geha RS Schneider LC Ring J Fitzpatrick TB, Eisen AZ, Wolff K, Freedberg IM, Austen KF Atopic dermatitis (atopic eczema) Dermatology in General Medicine 1993 4 New York: McGraw Hill 1543 1563

[B6] Ring J Angewandte Allergologie, 3. Aufl 2004 München: MMV Medizin Verlag

[B7] Ring J Ruzicka T, Ring J, Przybilla B Atopy: condition, disease, or syndrome? Handbook of Atopic Eczema 2006 2 Berlin, Germany: Springer 3 9

[B8] Morren MA Przybilla B Bamelis M Atopic dermatitis: triggering factors J Am Acad Dermatol 1994 1 467 473 10.1016/S0190-9622(94)70213-6 8077475

[B9] Przybilla B Ring J Food allergy and atopic eczema Semin Dermatol 1990 1 220 225 2206923

[B10] Skov L Baadsgaard O Ultraviolet B-exposed major histocompatibility complex class II positive keratinocytes and antigen-presenting cells demonstrate a differential capacity to activate T cells in the presence of staphylococcal superantigens Br J Dermatol 1996 1 824 830 10.1111/j.1365-2133.1996.tb06310.x 8736320

[B11] van Bever HP Docx M Stevens WJ Food and food additives in severe atopic dermatitis Allergy 1989 1 588 594 10.1111/j.1398-9995.1989.tb04205.x 2610332

[B12] Barnetson RSTC MacFarlane HAF Benton EC House dust mite allergy and atopic eczema: a case report Br J Dermatol 1987 1 857 860 10.1111/j.1365-2133.1987.tb04905.x 3620346

[B13] Fukuda H Imayama S Okada T Mite-free room (MFR) for the management of atopic dermatitis Jpn J Allergol 1991 1 626 632 1892445

[B14] Ring J Brockow K Abeck D The therapeutic concept of "patient management" in atopic eczema Allergy 1996 1 206 215 8792916

[B15] Sanda T Yasue T Oohashi M Yasue A Effectiveness of house dust-mite allergen avoidance with atopic dermatitis J Allergy Clin Immunol 1992 1 653 657 10.1016/0091-6749(92)90370-H 1545086

[B16] Tan B Weald D Strickland I Friedman P Double-blind controlled trial of effect of housedust-mite allergen avoidance on atopic dermatitis Lancet 1996 1 15 18 10.1016/S0140-6736(96)91556-1 8531541

[B17] Tupker R DeMonchy J Coenraads P Homan A van der Meer J Induction of atopic dermatitis by inhalation of house dust mite J Allergy Clin Immunol 1996 1 1064 1070 10.1016/S0091-6749(96)70259-2 8626983

[B18] Grewe M Gyufko K Schöpf E Krutmann J Lesional expression of interferon-gamma in atopic eczema Lancet 1994 1 25 26 7905045 10.1016/s0140-6736(94)90879-6

[B19] Leung DYM Ghan AK Schneeberger EE Geha RS Characterization of the mononuclear cell infiltrate in atopic dermatitis using mononuclear antibodies J Allergy Clin Immunol 1983 1 47 56 10.1016/0091-6749(83)90546-8 6337197

[B20] Ohmen JD Hanifin JM Nickoloff BJ Overexpression of IL-10 in atopic dermatitis. Contrasting cytokine patterns with delayed-type hypersensitivity reactions J Immunol 1995 1 1956 1963 7836775

[B21] Renz H Jujo K Bradley K Enhanced IL-4 production and IL-4 receptor expression in atopic dermatitis and their modulation by interferon-gamma J Invest Dermatol 1992 1 403 408 10.1111/1523-1747.ep12616114 1401997

[B22] Sowden J Powell R Allen B Selective activation of circulating CD4^+ ^lymphocytes in severe adult atopic dermatitis Br J Dermatol 1992 1 228 232 10.1111/j.1365-2133.1992.tb00119.x 1390166

[B23] Vercelli D Jabara H Lauener R Geha R IL-4 inhibits the synthesis of INF-gamma and induces the synthesis of IgE in human mixed lymphocyte cultures J Immunol 1990 1 570 573 2136895

[B24] Schäfer L Kragballe K Abnormalities in epidermal lipid metabolism in patients with atopic dermatitis J Invest Dermatol 1991 1 10 15 10.1111/1523-1747.ep12514648 1987285

[B25] Maeda K Yamamoto K Tanaka Y Anan S Yoshida H House dust mite (HDM) antigen in naturally occurring lesions of atopic dermatitis (AD): the relationship between HDM antigen in the skin and HDM antigen-specific IgE antibody J Dermatol Sci 1992 1 73 77 10.1016/0923-1811(92)90038-D 1599902

[B26] Bieber T de la Salle C Wollenberg A Hakimi J Chizzonite R Ring J Constitutive expression of the high affinity receptor for IgE (FCeR1) on human Langerhans-cells J Exp Med 1992 1 1285 1290 10.1084/jem.175.5.1285 1533242 PMC2119213

[B27] Bieber T Rieger A Neuchrist C Induction of FCeR2/CD23 on human epidermal Langerhans cells by human recombinant IL4 and IFN J Exp Med 1989 1 309 314 10.1084/jem.170.1.309 2526195 PMC2189378

[B28] Bieber T FCeRI on human Langerhans cells: a receptor in seach of new functions Immunol Today 1994 1 52 53 10.1016/0167-5699(94)90132-5 8155262

[B29] Bruijnzeel-Koomen C van Wichen DF Toonstra J Berrens L Bruijnzeel PLB The presence of IgE molecules on epidermal Langerhans cells in patients with atopic dermatitis Arch Dermatol Res 1986 1 199 205 10.1007/BF00412924 2942117

[B30] Maurer D Ebner C Reininger B Fiebiger E Kraft D Kinet Jp Stingl G The high affinity IgE receptor mediates IgE-dependent allergen presentation J Immunol 1995 1 6285 6290 7759866

[B31] Darsow U Ring J Etablierte Diagnostikverfahren Neurodermitis 1998 Landsberg: Ecomed 61 73

[B32] Ring J Kunz B Bieber T Vieluf D Przybilla B The "atopy patch test" with aeroallergens in atopic eczema J Allergy Clin Immunol 1989 1 195 [abstract]

[B33] Rostenberg A Sulzberger MD Some results of patch tests Arch Dermatol 1937 1 433 454 10.1001/archderm.1937.01470210059006

[B34] Mitchell E Chapman M Pope F Crow J Jouhal S Platts-Mills T Basophils in allergen-induced patch test sites in atopic dermatitis Lancet 1982 1 127 130 6119511 10.1016/s0140-6736(82)90379-8

[B35] Adinoff A Tellez P Clark R Atopic dermatitis and aeroallergen contact sensitivity J Allergy Clin Immunol 1988 1 736 742 10.1016/0091-6749(88)91047-0 3356851

[B36] Clark R Adinoff A Aeroallergen contact can exacerbate atopic dermatitis: patch test as a diagnostic tool J Am Acad Dermatol 1989 1 863 869 10.1016/S0190-9622(89)70269-3 2600213

[B37] Imayama S Hashizuma T Miyahara H Combination of patch test and IgE for dust mite antigens differentiates 130 patients with atopic dermatitis into four groups J Am Acad Dermatol 1992 1 531 538 10.1016/0190-9622(92)70218-5 1401304

[B38] Platts-Mills T Mitchell E Rowntree S Chapman M Wilkins S The role of dust mite allergens in atopic dermatitis Clin Exp Dermatol 1983 1 233 247 10.1111/j.1365-2230.1983.tb01776.x 6883791

[B39] Reitamo S Visa K Kaehoenen K Käykhö A Lauerna I Stubb S Salo OP Patch test reactions to inhalant allergens in atopic dermatitis Acta Derm Venereol 1989 1 119 121 2477976

[B40] Reitamo S Visa K Kähönen K Stubb S Salo OP Eczematous reactions in atopic patients caused by epicutaneous testing with inhalant allergens Br J Dermatol 1986 1 303 309 10.1111/j.1365-2133.1986.tb02821.x 3513815

[B41] Seidenari S Manzini BM Danese P Giannetti A Positive patch tests to whole mite culture and purified mite extracts in patients with atopic dermatitis, asthma and rhinitis Ann Allergy 1992 1 201 206 1524275

[B42] Seidenari S Manzini M Danese P Patch testing with pollens of Gramineae in patients with atopic dermatitis and mucosal atopy Contact Dermatitis 1992 1 125 126 10.1111/j.1600-0536.1992.tb05232.x 1395623

[B43] Seifert H Wollemann G Seifert B Borelli S Neurodermitis: Eine Protein-Kontaktdermatitis? Dtsch Derm 1987 1 1204 1214

[B44] Vieluf D Kunz B Bieber T Przybilla B Ring J Atopy patch test with aeroallergens in patients with atopic eczema Allergo J 1993 1 9 12

[B45] Vocks E Seifert H Seifert B Drosner M Ring J, Przybilla B Patch test with immediate type allergens in patients with atopic dermatitis New Trends in Allergy III 1991 Berlin: Springer 230 233

[B46] Gondo A Saeki N Tokuda Y Challenge reactions in atopic dermatitis after percutaneous entry of mite antigen Br J Dermatol 1986 1 485 493 10.1111/j.1365-2133.1986.tb06243.x 3778816

[B47] Norris P Schofield O Camp R A study of the role of house dust mite in atopic dermatitis Br J Dermatol 1988 1 435 440 10.1111/j.1365-2133.1988.tb02440.x 3281704

[B48] Bruijnzeel-Koomen C van Wichen D Spry C Venge P Bruynzeel P Active participation of eosinophils in patch test reactions to inhalant allergens in patients with atopic dermatitis Br J Dermatol 1988 1 229 238 10.1111/j.1365-2133.1988.tb01779.x 3348968

[B49] Langeland T Braathen L Borch M Studies of atopic patch tests Acta Derm Venereol 1989 1 105 109 10.2340/00015555144105109 2529724

[B50] van Voorst Vader PC Lier JG Woest TE Coenraads PJ Nater JP Patch tests with house dust mite antigens in atopic dermatitis patients: methodological problems Acta Derm Venereol 1991 1 301 305 1681645

[B51] Tanaka Y Anan S Yoshida H Immunohistochemical studies in mite antigen-induced patch test sites in atopic dermatitis J Dermatol Sci 1990 1 361 368 10.1016/0923-1811(90)90593-3 2073493

[B52] Ring J Bieber T Vieluf D Kunz B Przybilla B Atopic eczema, Langerhans cells and allergy Int Arch Allergy Appl Immunol 1991 1 194 201 10.1159/000235361 1937874

[B53] Andersen KE The European Environmental and Contact Dermatitis Research Group. Contact dermatitis: a review Contact Dermatitis 1987 1 55 78 10.1111/j.1600-0536.1987.tb01382.x 3552400

[B54] Fisher AA Contact Dermatitis 1986 3 Philadelphia: Lea & Febiger 686 691

[B55] Darsow U Vieluf D Ring J Atopy patch test with different vehicles and allergen concentrations--an approach to standardization J Allergy Clin Immunol 1995 1 677 684 10.1016/S0091-6749(95)70172-9 7897150

[B56] Darsow U Vieluf D Ring J The atopy patch test: an increased rate of reactivity in patients who have an air-exposed pattern of atopic eczema Br J Dermatol 1996 1 182 186 10.1111/j.1365-2133.1996.tb01144.x 8881657

[B57] Darsow U Vieluf D Ring J for the APT study group Evaluating the relevance of aeroallergen sensitization in atopic eczema using the tool "atopy patch test": a randomized, double-blind multicenter study J Am Acad Dermatol 1999 1 187 193 10.1016/S0190-9622(99)70186-6 10025743

[B58] Darsow U Vieluf D Berg B Dose response study of atopy patch test in children with atopic eczema Pediatr Asthma Allergy Immunol 1999 1 115 122 10.1089/pai.1999.13.115

[B59] Darsow U Laifaoui J Bolhaar S The prevalence of positive reactions in the atopy patch test with aeroallergens and food allergens in subjects with atopic eczema: a European multicenter study Allergy 2004 1 1318 1325 10.1111/j.1398-9995.2004.00556.x 15507101

[B60] Kerschenlohr K Günther S Darsow U Ollert M Wollenberg A Clinical and immunological reactivity to aeroallergens in "intrinsic" atopic dermatitis patients J Allergy Clin Immunol 2003 1 195 197 10.1016/S0091-6749(03)70068-2 12532120

[B61] Darsow U Behrendt H Ring J Gramineae pollen as trigger factors of atopic eczema: evaluation of diagnostic measures using the atopy patch test Br J Dermatol 1997 1 201 207 10.1046/j.1365-2133.1997.18061889.x 9292067

[B62] Wistokat-Wülfing A Schmidt P Darsow U Ring J Kapp A Werfel T Atopy patch test reactions are associated with T lymphocyte-mediated allergen-specific immune responses in atopic dermatitis Clin Exp Allergy 1999 1 513 521 10.1046/j.1365-2222.1999.00510.x 10202366

[B63] European Task Force on Atopic Dermatitis. Severity scoring of atopic dermatitis: the SCORAD index Dermatology 1993 1 23 31 10.1159/000247298 8435513

[B64] Ramb-Lindhauer CH Feldmann A Rotte M Neumann CH Characterization of grass pollen reactive T-cell lines derived from lesional atopic skin Arch Dermatol Res 1991 1 71 76 10.1007/BF00371611 1712577

[B65] Sager N Feldmann A Schilling G Kreitsch P Neumann C House dust mite-specific T cells in the skin of subjects with atopic dermatitis: frequency and lymphokine profile in the allergen patch test J Allergy Clin Immunol 1992 1 801 810 10.1016/0091-6749(92)90434-4 1373161

[B66] van Reijsen FC Bruynzeel-Koomen CAFM Kalthoff FS Maggi E Romagnani S Westland JKT Mudde GC Skin-derived aeroallergen-specific T-cell clones of T_H_2 phenotype in patiens with atopic dermatitis J Allergy Clin Immunol 1992 1 184 192 10.1016/0091-6749(92)90070-I 1380019

[B67] Weissenbacher S Bacon T Targett D Behrendt H Ring J Darsow U Atopy patch test--reproducibility and elicitation of itch in different application sites Acta Derm Venereol 2005 1 147 151 10.1080/00015550410024418 15823910

[B68] Lau S Ehnert B Cremer B Nasert S Büttner P Czarnetzki BM Wahn U Häusliche Milbenallergenreduktion bei spezifisch sensibilisierten Patienten mit atopischem Ekzem Allergo J 1995 1 432 435

[B69] Darsow U Ring J Airborne and dietary allergens in atopic eczema: a comprehensive review of diagnostic tests Clin Exp Dermatol 2000 1 544 551 10.1046/j.1365-2230.2000.00695.x 11122226

[B70] Weissenbacher S Bacon T Targett D Behrendt H Ring J Darsow U Atopy patch test--reproducibility and elicitation of itch in different application sites Acta Derm Venereol 2005 1 147 151 10.1080/00015550410024418 15823910

[B71] Weissenbacher S Traidl-Hoffmann C Eyerich K Modulation of atopy patch test and skin prick test by pretreatment with 1% pimecrolimus cream Int Arch Allergy Immunol 2006 1 239 244 10.1159/000093249 16691030

[B72] Turjanmaa K Darsow U Niggemann B Rancé F Vanto T Werfel T EAACI/GA2LEN Position Paper: present status of the atopy patch test--position paper of the Section on Dermatology and the Section on Pediatrics of the EAACI Allergy 2006 1 1377 1384 10.1111/j.1398-9995.2006.01136.x 17073865

[B73] Wüthrich B Neurodermitis atopica sive constitutionalis. Ein pathogenetisches Modell aus der Sicht des Allergologen Akt Dermatol 1983 1 1 7

[B74] Johansson SGO Bieber T Dahl R Revised nomenclature for allergy for global use: report of the Nomenclature Review Committee of the World Allergy Organization, October 2003 J Allergy Clin Immunol 2004 1 832 836 10.1016/j.jaci.2003.12.591 15131563

